# Distal Biceps and Triceps Injuries

**DOI:** 10.2174/1874325001711011364

**Published:** 2017-11-30

**Authors:** James C. Beazley, Thomas M. Lawrence, Steven J. Drew, Chetan S. Modi

**Affiliations:** Coventry and Warwickshire Shoulder and Elbow Unit, University Hospitals Coventry and Warwickshire NHS Trust, Coventry, CV2 2DX, UK

**Keywords:** Distal biceps, Distal triceps, Elbow joint, Reconstruction, Tendon rupture

## Abstract

**Background:**

Rupture of the distal biceps and triceps tendons are relatively uncommon injuries typically occurring in middle-aged males as a result of eccentric loading of the tendon.

**Methods:**

A literature search was performed and the authors’ personal experiences reported.

**Results:**

This review discusses the diagnosis, indications and guidelines for management of these injuries and provides a description of the authors’ preferred operative techniques.

**Conclusion:**

Whilst non-operative treatment may be appropriate for patients with low functional demands, surgical management is the preferred option for the majority of patients. We have described a cortical button technique and osseous tunnel technique utilised at our institution for distal biceps and triceps tendon fixation respectively. For biceps or triceps tendon injuries, those receiving an early diagnosis and undergoing surgical intervention, an excellent functional outcome can be expected.

## 1 INTRODUCTION

### 1.1 Distal Biceps Tendon Rupture

#### 1.1.1 Aetiology and Anatomy

Rupture of the distal biceps tendon occurs with an incidence of 1.2 in 100,000 [1]. Ninety percent of patients are male with an average age of 47 years [1]. Injury usually occurs in a weakened tendon during eccentric loading of the elbow. At the time of rupture the elbow is typically held at 90 degrees flexion and full supination [2]. Weakening of the tendon is multifactorial but both smoking and steroids have been shown to significantly increase the risk of tendon rupture [1] probably through a mechanism of reduced vascularity. Other independent risk factors include male sex and bodybuilding [1]. Repeated mechanical impingement during supination and pronation is also thought to contribute towards tendon rupture [3].

Until recently, it was believed that the two proximal heads of the biceps merge at the level of the deltoid tuberosity and then function as a single unit [4]. Recent work by Eames *et al*. has suggested that there is often little interdigitation of the muscle bellies and although the tendons may fuse at the lacertus fibrosus, the tendons continue in line with their respective muscle bellies and can still be easily separated into their separate bundles [5]. The long head inserts onto the proximal part of the radial tuberosity and the short head inserts onto the distal part of the radial tuberosity. Both tendons insert approximately 24 degrees from the apex in the axial plane thus enabling the radial side of the tuberosity to act as a cam, increasing the turning moment of the biceps [6]. The centre of the long head is slightly posterior to the centre of the short head. The separate insertion points of the long and short heads facilitates different positions of maximal function with the short head being the stronger arm flexor and supinator in the pronated and neutral forearm whilst the long head with its slightly posterior insertion point can generate greater supination torque in the fully supinated forearm [7] thus facilitating a greater effective arc of supination.

#### 1.1.2 Clinical Presentation and Diagnosis

Patients typically describe a sudden, severe pain in the antecubital fossa on maximal eccentric loading, which may be accompanied by hearing a snap or pop. Patients may complain of a temporary pseudo-paralysis of the elbow or demonstrate pain and weakness on flexion and supination of the elbow against resistance. Bruising often presents over the medial aspect of the elbow, although this finding in not consistent, especially if the lacertus fibrosus is intact, thus confining the hematoma [8]. If the lacertus fibrosus has been ruptured there may be abnormal contour of the distal biceps due to the retraction [8, 9].

**Fig. (1):**
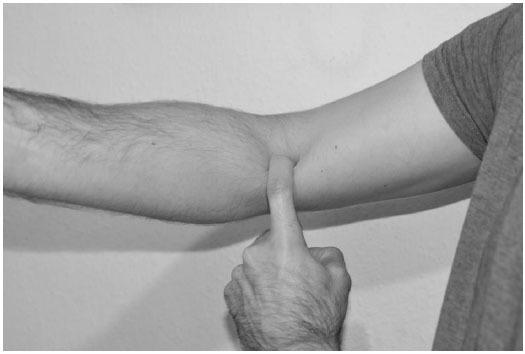
The hook test - when the biceps tendon is intact, as above, the examiner can fully insert the finger under the lateral edge of the biceps tendon. The finger passes between the biceps tendon and underlying brachialis muscle.

The ‘Hook’ test has been developed by O’Driscoll *et al* to confirm the diagnosis of distal biceps tendon rupture [10]. The patient is asked to flex the elbow to 90 degrees and fully supinate their forearm. The examiner’s index finger is brought in from the lateral aspect of the antecubital fossa to ‘hook’ the biceps tendon. If the tendon can be hooked and brought forwards this is an intact hook test (Fig. **1**). If the biceps tendon is completely avulsed there is no cord-like structure under which the examiner may hook their index finger and the hook test is “abnormal”. The lacertus fibrosis can also be examined. The lacertus fibrosus feels like a thin, sheet like structure on the medial side and may be avulsed in a complete tendon rupture. It can be differentiated from the biceps tendon as it is not fully ‘hookable’ although easily palpable from the medial side. A modification of the hook test has also been described for the diagnosis of partial tears in which the tendon is hooked and vigorously pulled forwards. If the tendon is hookable, but pulling the tendon produces pain this is suggestive of a partial tendon rupture. O’Driscoll reported a sensitivity and specificity of 100% for hook test for the diagnosis of complete distal biceps tendon ruptures in 33 patients with complete tears. In other centres, Devereaux and ElMaraghy have reported a sensitivity and specificity of 81% and 100% respectively for the hook test in the diagnosis of complete distal biceps tendon rupture [11].

Given the high specificity of the hook test, imaging is generally not required for patients with a positive test for complete rupture. Patients with a positive modified test and patients with a negative hook test but a high clinical suspicion require further imaging to confirm the diagnosis of partial tear. Both ultrasonography (US) and magnetic resonance imaging (MRI) can be used to confirm the diagnosis of a partial tear. A literature review generated no high level evidence to suggest superiority of one modality over the other however MRI is considered to be the gold standard [11] with O’Driscoll reporting a sensitivity and specificity for MRI of 92% and 85% respectively [10].

## 2 TREATMENT

### 2.1 Non-Operative

Complete distal biceps tendon rupture can be managed non-operatively, however most series report a significant reduction in supination strength and endurance with some series also reporting a reduction in flexion strength. In the largest reported series of conservatively treated patients, Freedman *et al*. reported a 37% reduction (p<0.05) in supination strength but a non-significant reduction in flexion strength [12]. Morrey **et al*.* reported a 40% reduction in supination and a 31% reduction in flexion strength in a smaller series of non-operatively treated patients [13]. Baker and Bierwagen reported an average difference in supination and flexion strength of 27% and 21% respectively in addition to a decrease in endurance [14]. Patients who select conservative treatment should be informed that they are likely to experience a reduction in supination strength and endurance and may also experience a reduction in flexion strength. Non-operative treatment is therefore usually reserved for patients with low functional demands or those that have significant risk factors for peri-operative complications.

### 2.2 Surgical Management of Complete Tears

The distal biceps tendon can be repaired via a single incision or via a two-incision technique. Dobbie developed the original one incision technique, however a high rate of radial nerve injury was reported [15] which lead to the two-incision technique being developed by Boyd and Anderson [16]. A recent systematic review by Watson *et al*. reported a complication rate of 23.9% (78 of 327 elbows) for one-incision procedures and 25.7% (44 of 171 elbows) for two-incision procedures (p = 0.32) [17]. With respect to functional outcome there is no clear benefit of one technique over the other. In the only randomised controlled trial examining the difference between single and two incision techniques Grewal *et al*. reported no difference in their primary outcome measure (American Shoulder and Elbow Surgeons (ASES) elbow score) at one year^18^.

Numerous fixation methods have been developed. These include bone tunnels, suture anchors, intraosseous screws and suspensory cortical buttons. Watson reported a complication rate of 26.4% (seventy-five of 284) for suture anchors, 20.4% (thirty-four of 167) for bone tunnels, 44.8% (thirteen of twenty-nine) for intraosseous screws, and 0% (zero of eighteen) for cortical button fixation (p = 0.03) [17]. At our institution we used cortical button fixation, which is described below.

### 2.3 Surgical Technique

**Fig. (2):**
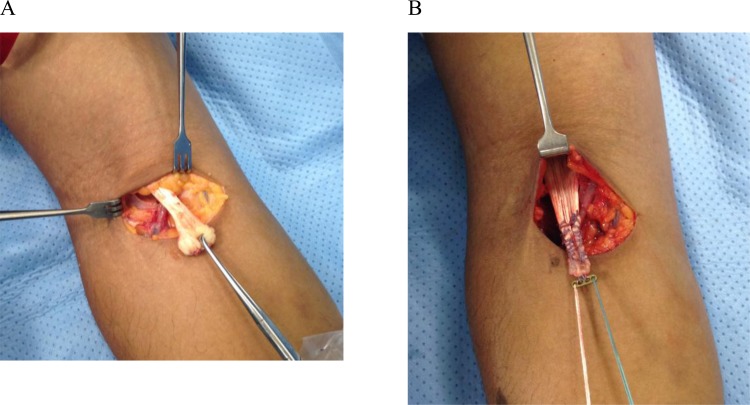
A: retrieval of tendon through transverse incision, taking care to identify and protect the lateral cutaneous nerve of the forearm. B: retrieved tendon is whip stitched to endobutton with 2 Orthocord.

**Fig. (3):**
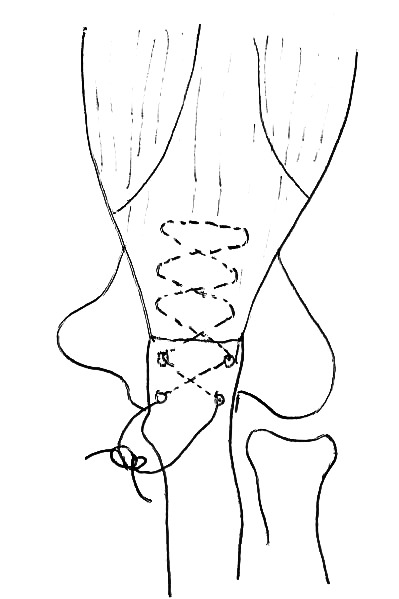
Endobutton fixation of distal biceps. Tendon is whip stitched to Endobutton with 2 Orthocord and a 4.5mm and 7mm hole drilled in far and near cortex of radius respectively (A). Endobutton with attached tendon is passed through radius (B). Endobutton flipped (C).

**Fig. (4):**
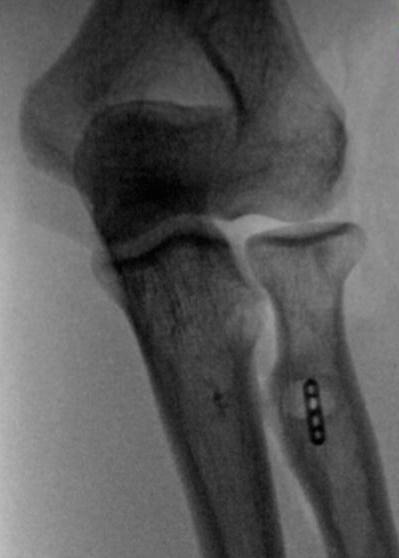
X-ray confirming correct position of cortical button at level of bicipital tuberosity.

Patients are positioned supine and a transverse incision is made in the anterior elbow crease at the level of the radial tuberosity. Care is taken to identify and protect the lateral cutaneous nerve of the forearm. Blunt dissection is used to retrieve the torn biceps tendon proximal to the incision (Fig. **2A**). The elbow is flexed and the tendon end is delivered out of the wound and prepared with a number 2 Orthocord locking stitch for at least 2cm, also incorporating the cortical button (Fig. **2B**). Blunt dissection is then used to identify the radial tuberosity. Recurrent radial artery branches often require ligation to allow access to the tuberosity. Hand held retractors are used to expose the tuberosity as placement of lever retractors behind the radial neck may lead to posterior interosseous nerve injury. With the forearm fully supinated, a beath pin is drilled through the tuberosity and is bicortically over-drilled with a 4.5mm cannulated drill to create a foramen for the cortical button (Fig. **3**). The radius is then unicortally over-drilled (usually 7mm) with an appropriately sized reamer creating a unicortical foramen for the tendon. The button is pulled through both cortices and deployed. We ensure full deployment of the button has occurred with the use of fluoroscopic imaging (Fig. **4**).

We keep patients in a sling for the first week to protect the wound. Following the first week we allow gentle active-assisted motion of the elbow. Resistance exercises are commenced after 6 weeks and full activities including heavy lifting and sports are permitted after 3 months.

### 2.4 Delayed Presentation

Presentation beyond three weeks leads to musculotendinous retraction and makes anatomic repair a challenge. Historically delayed presentations were managed with either tenodesis of the biceps tendon to brachialis or tendon reconstruction. Whilst studies have shown good recovery of flexion power with brachialis tenodesis, supination power is substantially reduced [19]. The biceps motor tendon unit has been shown to gradually stretch following repair which, combined with improvements in pull out strength of fixation devices has replaced the need for tendon reconstruction in most cases. Morrey *et al* reported the results of 23 cases in which the biceps were repaired in extreme flexion with good results and concluded contracted distal biceps tendons may be reliably reattached to their anatomic insertion with up to 90° of elbow flexion [20]. For tendons that have retracted such that reattachment at 90^o^ of elbow flexion is not possible tendon reconstruction using auto-graft or allograft is possible. Donor sites include Achilles tendon, semitendinosus, fascia lata, and flexor carpi radialis [8]. All report similar outcomes but direct comparison is difficult due to small numbers reported.

### 2.5 Surgical Management of Partial Tears

There is considerable controversy regarding the management of partial tears. For tears involving less than 50% of the footprint there is general agreement that non-operative management should be pursued [21-23]. For tears involving more than 50% of the footprint some authors advocate complete division and reattachment [18-20] however, the natural history of partial tendon tears has been poorly reported.

## 3 TRICEPS TENDON RUPTURE

### 3.1 Aetiology and Anatomy

Triceps tendon rupture is an extremely rare injury. Anzel reviewed the Mayo clinic experience of upper limb tendon rupture and found less than 1% of all tendon ruptures were triceps ruptures [24]. Triceps tendon injuries typically occur as a result of a fall onto an outstretched hand resulting in eccentric lengthening of the contracting triceps tendon [8] however injury as a result of direct blow and laceration has also been reported [25]. The tear most commonly occurs at the triceps insertion site onto the olecranon however reports of apophyseal separation in adolescents also exist [26]. Tears in the musculotendinous junction and muscle belly are very uncommon [25]. Chronic renal failure, endocrine disorders, metabolic bone diseases as well as steroid use have been suggested as risk factors [27, 28] however owing to the low prevalence of triceps tendon rupture these factors cannot be defined as causative [8].

The triceps brachii is composed of three muscle bellies. The long head arises off the infraglenoid tubercle of the scapula, the medial head off the posterior aspect of the humerus distal to the spiral groove and the lateral head off the lateral intermuscular septum and the posterolateral aspect of the humerus above the spiral groove [4]. The triceps inserts as a bilaminate tendon over a wide area onto the tip of the olecranon with the insertional footprint averaging 466mm^2^ [29]. The medial insertion of the triceps is along the crest of the olecranon with the lateral insertion fanning out over the extensor carpi ulnaris, anconeus and also into the antebrachial fascia of the forearm [30].

### 3.2 Clinical Presentation and Diagnosis

Patients typically present complaining of pain on the posterior aspect of the elbow following suitable mechanism of injury. Swelling, tenderness and ecchymosis are identified on physical examination. A palpable gap may be identified just proximal to the olecranon, although this may be masked by swelling in the acute period. Some patients may not notice weakness of elbow extension as anconeus, aided by gravity, can substitute for the triceps, especially for activities below shoulder height [8]. Even with the action of anconeus, active extension against resistance will be limited or absent in cases of complete tears. A modification of the Thompson test has been developed to aid diagnosis. The patient is positioned prone with the elbow flexed and the forearm over the side of the table. The triceps bulk is then squeezed with an intact tendon generating some extension of the elbow [30].

Plain films may demonstrate the presence of an avulsion fracture of the olecranon known as the fleck sign [31]. Additionally, plain films are important in excluding associated injuries such as radial head fractures and medial collateral ligament avulsion [32]. As for distal biceps rupture, the diagnosis of complete triceps tendon injury can be made clinically. MRI and US may however be ordered to clarify whether there is a complete or a partial tendon tear [33].

## 4 TREATMENT

### 4.1 Non-Operative

As with distal biceps tendon rupture, patients with low functional demands can be managed non-operatively, accepting the weakness in extension strength associated with the injury.

There is controversy regarding the optimum management of partial tears. There have been studies reporting good results of non-operative treatment even in patients with high functional demands - most of these tears are therefore treated non-surgically unless the symptoms persist after three to six months of treatment [34].

### 4.2 Surgical Management of Complete Tears

Early primary repair is indicated for complete acute ruptures. Surgery is preferably preformed within the first few weeks of injury. Numerous case series have described a variety of techniques including the use of suture anchors [36], sutures through olecranon bone tunnels [25] and the use of k-wires and tension bands in cases with large avulsed olecranon bone fragments [34]. Owing to the low incidence of this condition no comparative data exists to evaluate the efficacy of one technique over another [35].

**Fig. (5):**
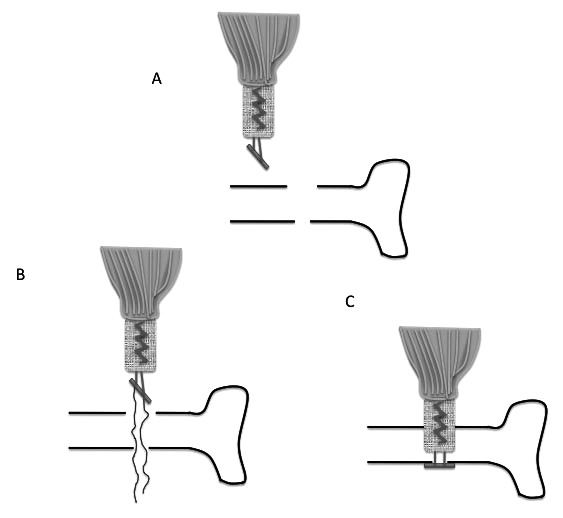
Repair of ruptured triceps tendon to bone with 2 Orthocord through 2.5mm drill holes.

At our institution we employ a bone tunnel technique. A No. 2 Orthocord suture is inserted through the ruptured triceps tendon using a Bunnell stitch technique. (Fig. **5**). Oblique drill holes are drilled with a 2.5mm drill. The two ends of the Ethibond are passed through the osseous tunnels and secured on the dorsal aspect of the proximal ulna.

The management of chronic tendon ruptures is a challenge. Occasionally, it is possible to repair the tendon using the technique described above. For chronic ruptures with significant tendon retraction, we use a technique described by Morrey, utilising a Achilles tendon graft and anconeus muscle flap [36].

Our postoperative rehabilitation protocol consists of a primary immobilisation in 30-60 degrees of elbow flexion for two weeks. We permit passive range of motion after the two weeks and active range of motion exercises at six weeks. Patients are advised that complete return to previous activities is not expected until at least three months post surgery.

## SUMMARY

Rupture of the distal biceps and triceps tendon are relatively uncommon injuries typically occurring in middle-aged males as result of eccentric loading of the tendon. Whilst non-operative treatment may be appropriate for patients with low functional demands, surgical management is the preferred option for the majority of patients. We have described a cortical button technique and osseous tunnel technique utilised at our institution for distal biceps and triceps tendon fixation respectively. For biceps or triceps tendon injuries, an excellent functional outcome can be expected for those receiving an early diagnosis and undergoing surgical intervention.
